# Longitudinal Blood-Based Biomarkers and Clinical Progression in Subjective Cognitive Decline

**DOI:** 10.1001/jamanetworkopen.2025.45862

**Published:** 2025-12-03

**Authors:** Calvin Trieu, Argonde C. van Harten, Mardou S. S. A. van Leeuwenstijn, Lisa-Marie Schlüter, Lynn Boonkamp, Azzam Aladdin, Sietske A. M. Sikkes, Elsmarieke van de Giessen, Inge M. W. Verberk, Charlotte E. Teunissen, Wiesje M. van der Flier

**Affiliations:** 1 Alzheimer Center Amsterdam, Department of Neurology, Amsterdam UMC, Vrije Universiteit Amsterdam, Amsterdam, the Netherlands; 2 Amsterdam Neuroscience, Neurodegeneration, Amsterdam, the Netherlands; 3 Neurochemistry Laboratory, Department of Clinical Chemistry, Amsterdam Neuroscience, Amsterdam, Netherlands; 4 Faculty of Behavioural and Movement Sciences, Department of Clinical, Neuro and Developmental Psychology & Clinical Neuropsychology, Vrije Universiteit Amsterdam, Amsterdam, the Netherlands; 5 Department of Radiology and Nuclear Medicine, Amsterdam Neuroscience, Amsterdam UMC, Vrije Universiteit Amsterdam, Amsterdam, the Netherlands

## Abstract

**Importance:**

Blood-based biomarkers identify Alzheimer disease and hold promise for monitoring disease progression, even in the preclinical disease stages.

**Objective:**

To investigate longitudinal trajectories of blood-based biomarkers and association with cognitive decline and risk of progression in individuals with subjective cognitive decline (SCD).

**Design, Setting, and Participants:**

This prospective cohort study (Subjective Cognitive Impairment Cohort) of individuals with SCD evaluated at a memory clinic underwent biennial biomarker collection and annual cognitive assessment and diagnostic evaluation from January 1, 2005, to December 31, 2023, with follow-up through 2023.

**Exposure:**

Amyloid status was determined using positron emission tomography or cerebrospinal fluid. Plasma Aβ_42/40_, phosphorylated tau 217 (pTau217), glial fibrillary acidic protein (GFAP), and neurofilament light (NfL) were measured biennially.

**Main Outcomes and Measures:**

Cognitive trajectories in memory, attention, language, executive function, and global cognition and clinical progression to mild cognitive impairment or dementia.

**Results:**

A total of 298 individuals (mean [SD] age, 61.55 [8.08] years; 174 [58.4%] male) with SCD were included, of whom 80 were amyloid-positive (A+) and 218 were amyloid-negative (A−). Mean (SD) follow-up was 4.8 (2.6) years. Individuals with SCD A+ were older (mean [SD] age, 65.25 [7.14] years; 42 [52.5%] male) than those with SCD A− (mean [SD] age, 60.19 [8.00] years; 132 [60.6%] male). For pTau217, GFAP, and NfL, baseline levels were higher in the A+ group compared with A− group (estimates [SE] amyloid β, 1.11 [0.11], 0.69 [0.13], and 0.36 [0.10], respectively; *P* < .001 for all). Additionally, these biomarkers showed steeper increases over time in the A+ group than in A− group (estimates [SE] time × amyloid status β, 0.07 [0.02], *P* < .001; 0.07 [0.02], *P* < .001; and 0.05 [0.02], *P* = .005, respectively). Longitudinal increases in pTau217 and GFAP were associated with cognitive decline over time in all domains (β time × biomarker slope = −0.02 to −0.04). Longitudinal decreases in Aβ_42/40_ and increases in NfL were associated with cognitive decline in global cognition (β = 0.03 [0.01], *P* = .04) and language (β = 0.04 [0.02], *P* = .03), and increases in NfL were also associated with decline in global cognition (β = −0.02 [0.01], *P* = .004), language (β = −0.03 [0.01], *P* = .007), and executive functioning (β = −0.03 [0.01], *P* = .02). Steeper pTau217 slope was associated with progression from SCD to mild cognitive impairment or dementia (hazard ratio [HR], 3.6; 95% CI, 1.8-7.4 per 0.05 SD increase per year; C index, 0.89; 95% CI, 0.84-0.93), as were steeper GFAP slope (HR, 1.5 [95% CI, 1.0-2.2]; C index, 0.81 [95% CI, 0.73-0.88]) and steeper NfL slope (HR, 2.6 [95% CI, 1.3-5.2]; C index, 0.77 [95% CI, 0.69-0.85]). Aβ_42/40_ slope was not associated with progression.

**Conclusions and Relevance:**

This cohort study of individuals with SCD suggests that longitudinal plasma pTau217 and GFAP are suitable biomarkers for monitoring AD pathology. Their changes were associated with cognitive decline and clinical progression, supporting their potential utility for early intervention and disease monitoring.

## Key Points

**Question:**

Are longitudinal changes in blood-based biomarkers associated with cognitive decline and clinical progression in individuals with subjective cognitive decline (SCD)?

**Findings:**

In this cohort study of 298 individuals with SCD, steeper slopes of plasma phosphorylated tau 217 (pTau 217), glial fibrillary acidic protein (GFAP), and neurofilament light were associated with cognitive decline, whereas pTau217 and GFAP slopes were associated with progression to mild cognitive impairment or dementia. One in 5 of the initially biomarker-negative participants transitioned to a biomarker-positive status during follow-up.

**Meaning:**

This study’s results suggest that longitudinal changes in pTau217 and GFAP are promising blood-based biomarkers for early identification and monitoring of Alzheimer disease progression in individuals with SCD.

## Teaser

This cohort study of individuals with subjective cognitive decline examines whether longitudinal changes in blood-based biomarkers are associated with cognitive decline and clinical disease progression.

## Introduction

Alzheimer disease (AD) is a progressive neurodegenerative disorder characterized by abnormal accumulation of amyloid β and tau proteins. These pathological changes begin decades before clinical symptoms are evident.^1,2^ Having abnormal AD-related blood-based biomarkers during preclinical stages is associated with future cognitive decline, although less is known about their longitudinal evolution.^3,4^ Understanding the biological trajectories of the AD-related blood-based biomarkers, as well as their associations with cognitive decline and the risk of progression to mild cognitive impairment (MCI) or dementia, could substantially advance early-stage diagnostic and therapeutic strategies. Individuals with subjective cognitive decline (SCD), who report self-perceived cognitive deterioration in the absence of objective impairment, represent a key population for these investigations, with those presenting at memory clinics having a higher risk of progression to MCI or dementia compared with cognitively unimpaired individuals.^5,6,7^ Those with biomarker evidence of AD are particularly relevant because they are increasingly recognized as being at a transitional stage (stage 2) in the AD continuum, positioning them further along the disease course.^8^

Investigating biomarker trajectories over time requires repeated assessments, which is challenging with cerebrospinal fluid (CSF) and positron emission tomography (PET) biomarkers due to their invasiveness and high costs. Blood-based biomarkers offer a less invasive and expensive way to assess AD-related pathology, enabling easier repetition and greater feasibility for longitudinal monitoring.^3,9,10^ Blood-based biomarkers for AD include phosphorylated tau 217 (pTau217), reflecting both amyloid and tau pathology; amyloid-β_42/40_ ratio (Aβ_42/40_), reflecting amyloid pathology; glial fibrillary acidic protein (GFAP), reflecting reactive astrocytosis; and neurofilament light chain (NfL), reflecting neuroaxonal injury.^10,11,12,13^

Former longitudinal studies in preclinical stages have primarily focused on cognitively unimpaired individuals and rarely investigated associations between biomarker changes and clinical progression.^14,15^ We aimed to investigate the trajectories of blood-based biomarkers in individuals with SCD and their association with longitudinal clinical outcomes. Specifically, we investigated (1) whether longitudinal trajectories of blood-based biomarkers differ between individuals with abnormal amyloid status (A+) compared with those with normal amyloid status (A−), (2) whether these trajectories were associated with the longitudinal cognitive decline and risk of clinical progression to MCI or dementia, and (3) the frequency and clinical relevance of change from normal to abnormal biomarker status during follow-up.

## Methods

### Participants

We included 298 individuals with SCD from the Subjective Cognitive Impairment Cohort (SCIENCe) from our memory clinic at the Alzheimer Center Amsterdam, who were enrolled between January 1, 2005, and December 31, 2021, and followed up until 2023.^16^ Participants underwent a standardized baseline workup, including a neurologic and neuropsychological examination, laboratory testing, and either CSF or amyloid PET. Participants were labeled SCD in a consensus meeting, when there was an absence of objective impairment, and criteria were not met for MCI, dementia, major psychiatric disorders, or other neurologic diseases.^16^ As part of SCIENCe, participants underwent annual follow-up visits, including neuropsychological testing and diagnostic evaluation for progression to MCI, AD dementia, or other types of dementia. Repeated blood collection was performed every 2 years. Progression was defined as MCI or dementia per National Institute on Aging Alzheimer’s Association criteria,^17^ with progression time defined as the date of first diagnosis (MCI if it preceded dementia, otherwise dementia). The mean (SD) follow-up time for blood and cognitive assessments was 4.8 (2.6) years (maximum, 15.6 years), and the median (range) number of blood samples was 3 (2-5). All participants provided written informed consent. The study was approved by the Medical Ethical Committee of the Vrije Universiteit University and was in compliance with the ethical standards set by the Declaration of Helsinki of 1975.^18^ This study was reported following the Strengthening the Reporting of Observational Studies in Epidemiology (STROBE) guideline for cohort studies and the Standards for Reporting Diagnostic Accuracy Studies (STARD) guideline.

### Amyloid Status

Amyloid status at baseline was determined using amyloid PET (n = 206) or CSF (n = 92). Amyloid positivity was defined by a visually rated positive PET scan result, CSF test results with Innotest Aβ_42_ levels less than 813 pg/mL (corrected for drift), or an Elecsys pTau/Aβ_42_ ratio greater than 0.02. Details on PET and CSF analysis are given in the eAppendix in Supplement 1.

### Neuropsychological Assessment

Cognitive performance was assessed using a standardized neuropsychological test battery, covering 4 cognitive domains, including memory, language, attention, and executive functioning. A detailed description of the tests is provided in the eAppendix in Supplement 1. Raw test scores were standardized into *z* scores using baseline as a reference and were then combined into cognitive domains. The cognitive domain *z* scores were averaged to represent global cognition. Missing values were imputed using a multilevel multiple imputation approach (mice package in R) before calculating *z* scores, ensuring robust representation of each domain. Overall missing data were under 5% for each test, except for the Rivermead Behavioral Memory Test (20%) and letter fluency (14%).

### Blood-Based Biomarkers

Blood-based biomarkers were measured on the Simoa HD-X analyzer using the Neurology 4-plex E Kit (Quanterix) and the Simoa pTau217 Kit (Janssen), as detailed in the eAppendix in Supplement 1. Cut-offs for amyloid positivity for the biomarkers were previously calculated in a population without dementia^19^ and were bridged to the current measurements using Passing-Bablok regression (eFigure 1 in Supplement 1). Hence, the cut-offs applied were Aβ_42/40_ less than 0.054, pTau217 greater than 0.048 pg/mL, GFAP greater than 83.9 pg/mL, and NfL greater than 12.7 pg/mL. The intra-assay and interassay coefficients of variation were below 10% for all biomarkers. Biomarker values more than 3 SDs from the mean and suspected of measurement error were excluded from the analyses (1 value each for Aβ_42/40_, pTau217, and NfL). In total, 1 participant had missing values for Aβ_42/40_, 4 for pTau217, 1 for NfL, and 0 for GFAP.

### Statistical Analysis

Analyses were performed in RStudio software, version 2022.12.0 (R Project for Statistical Computing), using the lme4 (linear mixed models) and JMBayes2 packages (joint models). Demographic differences were tested with unpaired *t* tests or Pearson χ^2^ as appropriate. All biomarker values were *z* transformed, and all models were adjusted for age and sex. Separate linear mixed models (LMMs) with time as the predictor were used to analyze the trajectory of each blood-based biomarker. Individual biomarker slopes estimated from these models were subsequently used as predictors in further analyses, with all models including biomarker slopes adjusted for baseline levels to isolate the independent effect of the slope. All analyses were considered statistically significant at a 2-sided *P* < .05.

#### Association Between Amyloid Status and Blood-Based Biomarker Changes

We analyzed the associations between baseline amyloid status and blood-based biomarker changes at baseline and over time. For this analysis, we used LMMs, including terms for amyloid status, time, and their interaction.

#### Association Between Blood-Based Biomarkers and Cognition

We used LMMs to assess associations between baseline biomarker levels or their rate of change (slopes derived from prior LMMs) and cognition over time, separately for each cognitive domain, by including baseline values or slopes as predictors, along with time and their interactions, while adjusting for age, sex, and education level. To visualize this association, we categorized biomarker slopes into tertiles (low, medium, and high). In addition, we used time-varying LMMs leveraging all available longitudinal data to estimate the mean association between biomarker levels and cognition across the entire follow-up period.

#### Association Between Blood-Based Biomarkers and Progression

We assessed the prognostic value of the baseline and the slope of biomarkers for progression to MCI or dementia using Cox proportional hazards regression models. The hazard ratio (HR) for baseline biomarker values reflects the risk of progression per 1-SD increase at baseline, whereas the HR for biomarker slope reflects the risk associated with a 0.05-SD per year increase in rate of change, chosen to reflect a standardized annual change. The biomarker slopes in tertiles were used to visualize the association between the biomarker trajectories and the risk of progression. In addition, we used joint models to estimate the mean effect of each unit increase in biomarker levels on the risk of progression across the entire follow-up period.

Prognostic performance of biomarker slopes on progression was investigated beyond demographics (age, sex, and baseline Mini-Mental State Examination [MMSE] score) and baseline biomarker values, comparing (1) demographics only, (2) demographics plus baseline biomarkers, (3) demographics plus both baseline and slope, and (4) demographics plus most contributory biomarkers identified via elastic net regularization. The concordance index (C index) was used as the primary measure of prognostic accuracy. Elastic net regularization was applied within a Cox proportional hazards regression framework to identify the most relevant variables of progression to MCI or dementia, with 5-fold cross-validation used to select the optimal penalty parameter (λ). To compare models, we used a bootstrap resampling approach (5000 iterations) to compute 95% CIs.

#### Biomarker Status Change

For each biomarker, participants were classified into 5 trajectory groups based on biomarker cut-offs: stable negative (individuals who remained below the cut-off throughout the study), stable positive (remained above the cut-off), positive change (transitioned from below to above the cut-off), negative change (transitioned from above to below the cut-off), and intermittent change (fluctuated above and below the cut-off).^19^ Participant numbers for each group were counted per biomarker. We used LMMs to evaluate whether the positive change and stable positive groups exhibited greater cognitive decline than the stable negative (reference) group by including terms for biomarker status change, time, and their interaction. The negative change and intermittent change groups were excluded from these analyses due to limited sample size.

## Results

### Demographic Characteristics

We included 298 individuals (mean [SD] age, 61.55 [8.08] years; 174 [58.4%] male and 124 [41.6%] female), comprising 80 (26.8%) individuals with A+ and 218 (73.2%) with A−. Compared with the A− group, the A+ group was older (estimate [SD], 65.25 [7.14] vs 60.19 [8.00] years; *P* < .001) and had a higher rate of *APOE* ε4 carriers (60 [75.0%] vs 64 [29.4%]; *P* < .001), whereas sex distribution did not differ (Table 1). In the total group, pTau217 (estimate [SE] time β = 0.07 [0.01]), GFAP (estimate [SE] time β = 0.11 [0.01]), and NfL (estimate [SE] time β = 0.09 [0.01]; *P* < .001 for all) increased over time, whereas Aβ_42/40_ remained stable (eFigure 2 in Supplement 1). Individuals with A+ showed a steeper cognitive decline in global cognition (and all other cognitive domains) compared with individuals with A− (estimate [SE] time × amyloid status β = −0.09 [0.01], *P* < .001 (eTable 1 in Supplement 1). During follow-up, 33 participants (11.1%) progressed to MCI or dementia: 22 (7.4%) progressed to MCI, 7 (2.3%) progressed to MCI and subsequently to dementia, and 4 (1.3%) progressed directly to dementia.

**Table 1.** Characteristics of the Study Participants^a^

Characteristic	No. (%) of patients			*P* value
	Total (N = 298)	A+ (n = 80)	A− (n = 218)	
Age, mean (SD), y	61.55 (8.08)	65.25 (7.14)	60.19 (8.00)	<.001
Sex				
Female	124 (41.6)	38 (47.5)	86 (39.4)	.26
Male	174 (58.4)	42 (52.5)	132 (60.6)	
*APOE *ε4 carriership	124 (41.6)	60 (75.0)	64 (29.4)	<.001
Educational level, median (IQR)	6.0 (5.0-6.0)	6.0 (5.0-7.0)	6.0 (5.0-6.0)	.84
MMSE score, mean (SD)	28.45 (1.43)	28.40 (1.27)	28.47 (1.49)	.71
MoCA score, mean (SD)	26.04 (2.55)	26.00 (2.90)	26.06 (2.36)	.94
Family history dementia	166 (55.7)	51 (63.7)	115 (52.8)	.055
Family history psychiatry	77 (25.8)	20 (25.0)	57 (26.1)	>.99
Follow-up time, mean (SD), y	4.83 (2.58)	4.06 (1.97)	5.12 (2.72)	<.001
Lost to follow-up	27 (9.1)	8 (10.0)	19 (8.7)	.91
Clinical progression	33 (11.1)	27 (33.8)	6 (2.8)	<.001
Aβ_42/40_, mean (SD)	0.06 (0.01)	0.05 (0.01)	0.06 (0.01)	<.001
pTau217, mean (SD), pg/mL	0.04 (0.03)	0.07 (0.04)	0.04 (0.01)	<.001
GFAP, mean (SD), pg/mL	84.80 (45.24)	115.87 (54.55)	73.40 (35.08)	<.001
NfL, mean (SD), pg/mL	13.78 (8.54)	17.57 (8.68)	12.38 (8.08)	<.001

Abbreviations: A+, amyloid positive; A−, amyloid negative; Aβ_42/40_, amyloid-β_42/40 _ratio; GFAP, glial fibrillary acidic protein; MMSE, Mini-Mental State Examination; MoCA, Montreal Cognitive Assessment; NfL, neurofilament light chain; pTau217, phosphorylated tau 217.

^a^ Demographic differences between groups were analyzed using either unpaired *t* tests or Pearson χ^2^ tests based on the nature of the data. At baseline, values were missing for 244 individuals in the MoCA group, 7 in the *APOE* ε4 group, 1 in the Aβ_42/40_ group, 4 in the pTau217 group, and 1 in the NfL group.

### Association Between Amyloid Status and Blood-Based Biomarker Changes

**Figure 1.**
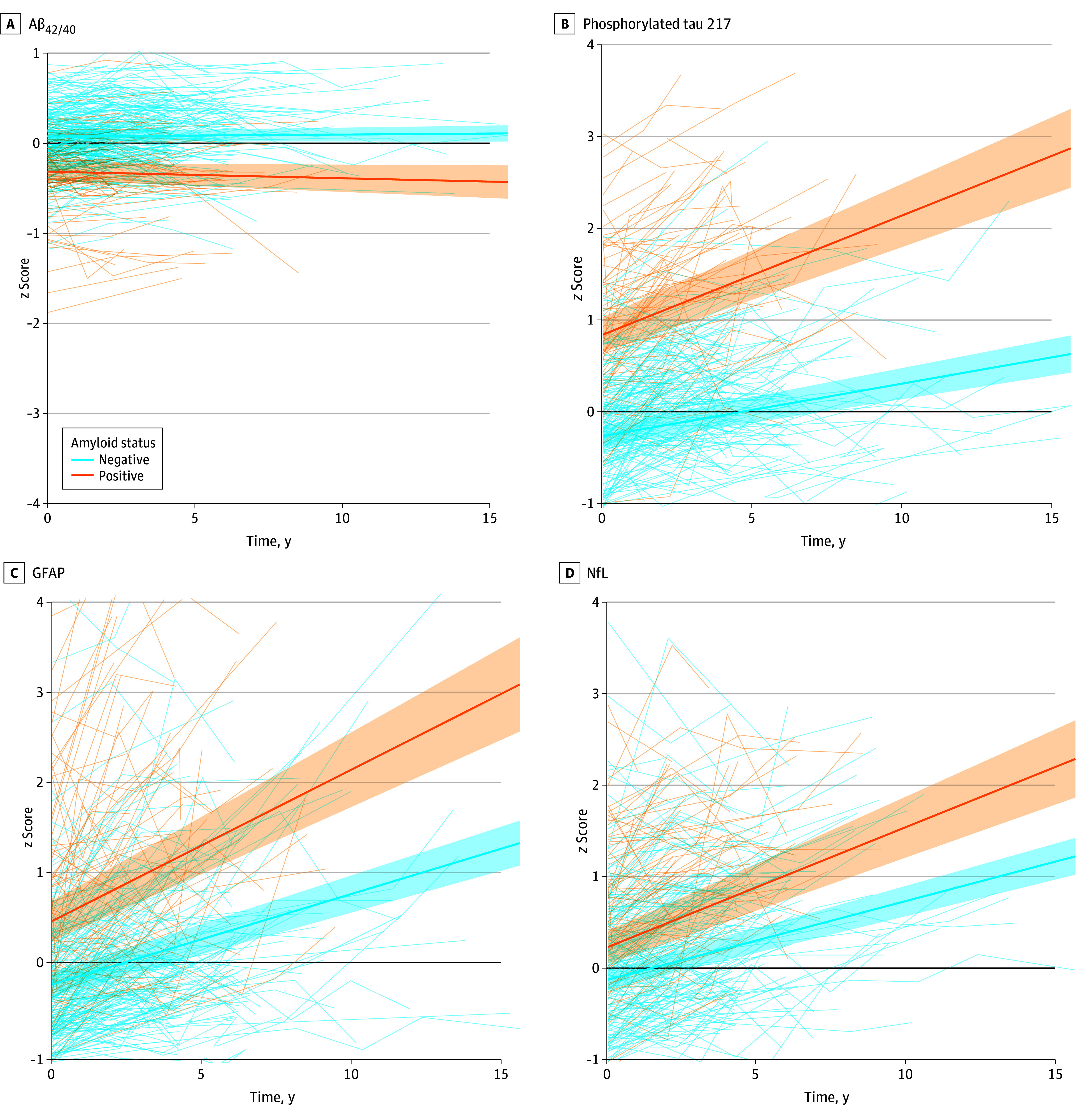
Association Between Amyloid Status and Blood-Based Biomarkers Over Time The figure shows the trajectories of blood-based biomarkers over time presented as *z* scores standardized to baseline biomarker values. The models were stratified by amyloid status and adjusted for age and sex, with values centered around the overall sample mean age and sex (mean age of 61.6 years and sex distribution of 58.4% male). Shaded areas indicate 95% CIs. Aβ_42/40_ indicates amyloid-β_42/40_ ratio; GFAP, glial fibrillary acidic protein; NfL, neurofilament light chain.

Individuals with A+ had a lower Aβ_42/40_ ratio at baseline compared with those with A− (estimate [SE] amyloid status β = −0.39 [0.05]; *P* < .001) (eTable 2 and eFigure 3 in Supplement 1), but there were no differences in rate of change between the A+ and A− groups (estimate [SE] time × amyloid status β = −0.01 [0.01]) (Figure 1). For pTau217, GFAP, and NfL, baseline levels were higher in the A+ group compared with A− group (1.11 [0.11], 0.69 [0.13], and 0.36 [0.10], respectively; *P* < .001 for all). Additionally, these biomarkers showed steeper increases over time in the A+ group than in the A− group (0.07 [0.02], *P* < .001; 0.07 [0.02], *P* < .001; and 0.05 [0.02], *P* = .005, respectively).

### Association Between Blood-Based Biomarkers and Cognition

**Figure 2.**
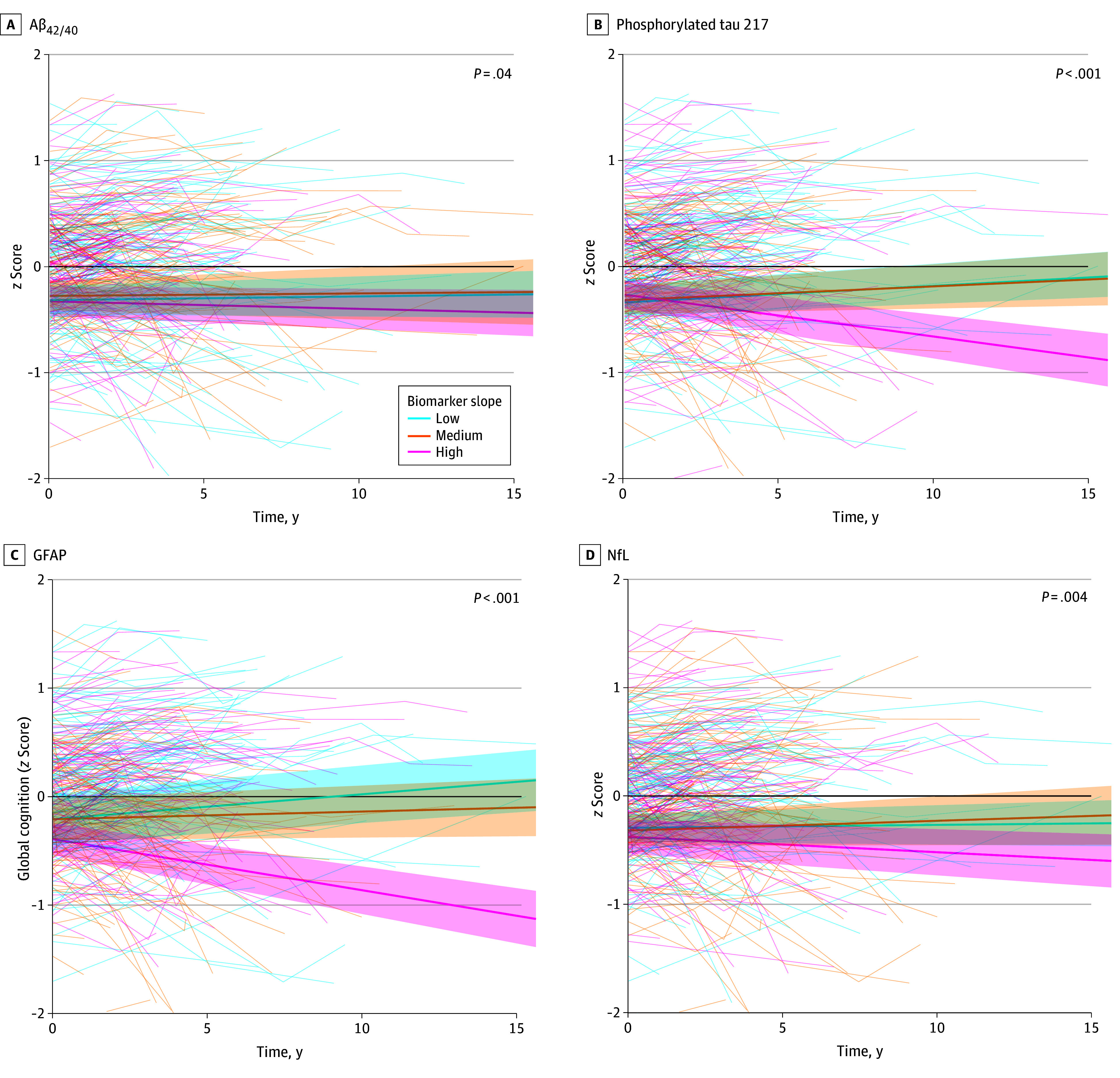
Associations Between Biomarker Slopes and Cognitive Trajectories Over Time The figure presents results from linear mixed models examining trajectories of cognitive domain scores (global cognition) over time in relation to biomarker slopes. Biomarker slopes were categorized into tertiles (low, medium, or high) for visualization. Cognitive scores are expressed as *z* scores. *P* values shown in the figure represent the biomarker × time interaction, derived from models using continuous biomarker slopes. Models were adjusted for baseline levels (and age, sex, and educational level) to isolate the independent effect of the slope over time. Shaded areas indicate 95% CIs. Aβ_42/40_ indicates amyloid-β_42/40_ ratio; GFAP, glial fibrillary acidic protein; NfL, neurofilament light chain.

LMMs with biomarker slopes as predictor, adjusted for baseline values, showed that steeper increases in pTau217 and GFAP were associated with cognitive decline over time across all domains (time × biomarker β = −0.05 to −0.03 for pTau217 and −0.03 to −0.02 for GFAP, *P* < .001 for all), whereas decreases in Aβ_42/40_ were only associated with cognitive decline in global cognition (β = 0.03 [0.01], *P* = .04) and language (β = 0.04 [0.02], *P* = .03), and increases in NfL were associated with decline in global cognition (β = −0.02 [0.01], *P*  = .004), language (β = −0.03 [0.01], *P* = .007), and executive functioning (β = −0.03 [0.01], *P* = .02) (Figure 2; eTable 3 and eFigure 4 in Supplement 1). Of note, baseline levels also demonstrated associations with cognitive decline (eTable 4 in Supplement 1). Data across all time points in time-varying LMMs showed that lower Aβ_42/40_ and higher pTau217, GFAP, and NfL were associated with worse cognitive performance across multiple domains (eTable 5 in Supplement 1).

### Association Between Blood-Based Biomarkers and Progression

Cox proportional hazards regression models revealed that lower baseline Aβ_42/40_ was associated with a higher risk of progression (HR, 4.09; 95% CI, 52.15-7.78), and adding it to the demographics model (age, sex, and baseline MMSE score) improved the C index from 0.69 (95% CI, 0.58-0.80) to 0.77 (95% CI, 0.68-0.85), indicating 77% prognostic accuracy for progression (Table 2; eFigure 5 in Supplement 1). In contrast, Aβ_42/40_ slope was not associated with risk and did not improve prognostic performance. Higher baseline pTau217 was associated with an increased risk (HR, 2.92; 95% CI, 2.07-4.11) and improved the C index from 0.69 (95% CI, 0.58-0.80) to 0.86 (95% CI, 0.79-0.91). pTau217 slope (0.05-SD yearly increase) was associated with a higher risk (HR, 3.61; 95% CI, 1.76-7.39), and adding it to the model showed further improvement to 0.89 (95% CI, 0.84-0.93). A higher baseline GFAP level was associated with a risk (HR, 1.78; 95% CI, 1.38-2.29) and improved the C index from 0.69 (95% CI, 0.58-0.80) to 0.77 (95% CI, 0.68-0.85). The GFAP slope was associated with a higher risk (HR, 1.51; 95% CI, 1.02-2.23) and showed a trend toward further improvement to 0.81 (95% CI, 0.73-0.88). A higher baseline NfL was associated with a higher risk (HR, 1.93; 95% CI, 1.37-2.73) and improved the C index from 0.69 (95% CI, 0.58-0.80) to 0.74 (95% CI, 0.64-0.84). The NfL slope was associated with a higher risk (HR, 2.56; 95% CI, 1.27-5.18) but did not further improve prognostic performance as indicated by the C index.

**Table 2.** Prognostic Performance of Blood-Based Biomarkers for Clinical Progression

Biomarker	HR (95% CI)^a^	*P* value	C index (95% CI)	C index change (95% CI)	
				Comparison with demographics^b^	Comparison with biomarker baseline^b^
Demographics	NR	NA	0.69 (0.58 to 0.80)	NA	NA
Aβ_42/40_ baseline	0.24 (0.13 to 0.47)	<.001	0.77 (0.68 to 0.85)	0.07 (0.02 to 0.15)	NA
Baseline	0.22 (0.12 to 0.43)	<.001	0.78 (0.68 to 0.86)	0.08 (0.02 to 0.17)	0.00 (−0.005 to 0.03)
Plus slope	0.73 (0.33 to 1.61)	.43			
pTau217 baseline	2.92 (2.07 to 4.11)	<.001	0.86 (0.79 to 0.91)	0.16 (0.07 to 0.27)	NA
Baseline	1.60 (1.01 to 2.53)	.047	0.89 (0.84 to 0.93)	0.19 (0.10 to 0.32)	0.03 (0.001 to 0.09)
Plus slope	3.61 (1.76 to 7.39)	<.001			
GFAP baseline	1.77 (1.38 to 2.29)	<.001	0.77 (0.68 to 0.85)	0.07 (0.01 to 0.17)	NA
Baseline	1.00 (0.55 to 1.83)	.10	0.81 (0.73 to 0.88)	0.11 (0.04 to 0.23)	0.04 (−0.00 to 0.10)
Plus slope	1.51 (1.02 to 2.23)	.04			
NfL baseline	1.93 (1.37 to 2.73)	<.001	0.74 (0.64 to 0.84)	0.04 (−0.002 to 0.13)	NA
Baseline	2.57 (1.69 to 3.91)	<.001	0.77 (0.69 to 0.85)	0.07 (0.01 to 0.18)	0.02 (−0.002 to 0.08)
Plus slope	2.56 (1.27 to 5.18)	.009			
Optimal combination	NR	NA	0.90 (0.86 to 0.94)	0.21 (0.11 to 0.33)	NA

Abbreviations: Aβ_42/40_, amyloid-β_42/40_ ratio; GFAP, glial fibrillary acidic protein; HR, hazard ratio; NA, not applicable; NfL, neurofilament light chain; NR, not reported; pTau217, phosphorylated tau 217.

^a^ The hazard ratio represents the risk for every 1 SD higher baseline biomarker value and for every 0.05 SD/y higher biomarker slope. For each biomarker, the first row (baseline) corresponds to a model including the baseline biomarker value alone, whereas subsequent rows (baseline and plus slope) correspond to models including both the baseline value and the longitudinal slope of the biomarker.

^b^ Two additional columns are included: comparison with demographics alone, which shows the median increase (95% CI) in prognostic performance (C index) for each specific model compared with the demographics-only model and comparison with biomarker baseline alone, which evaluates the median increase (95% CI) in prognostic performance for models that included the slope of the biomarker or combining baseline and slope relative to the baseline model of that specific biomarker.

**Figure 3.**
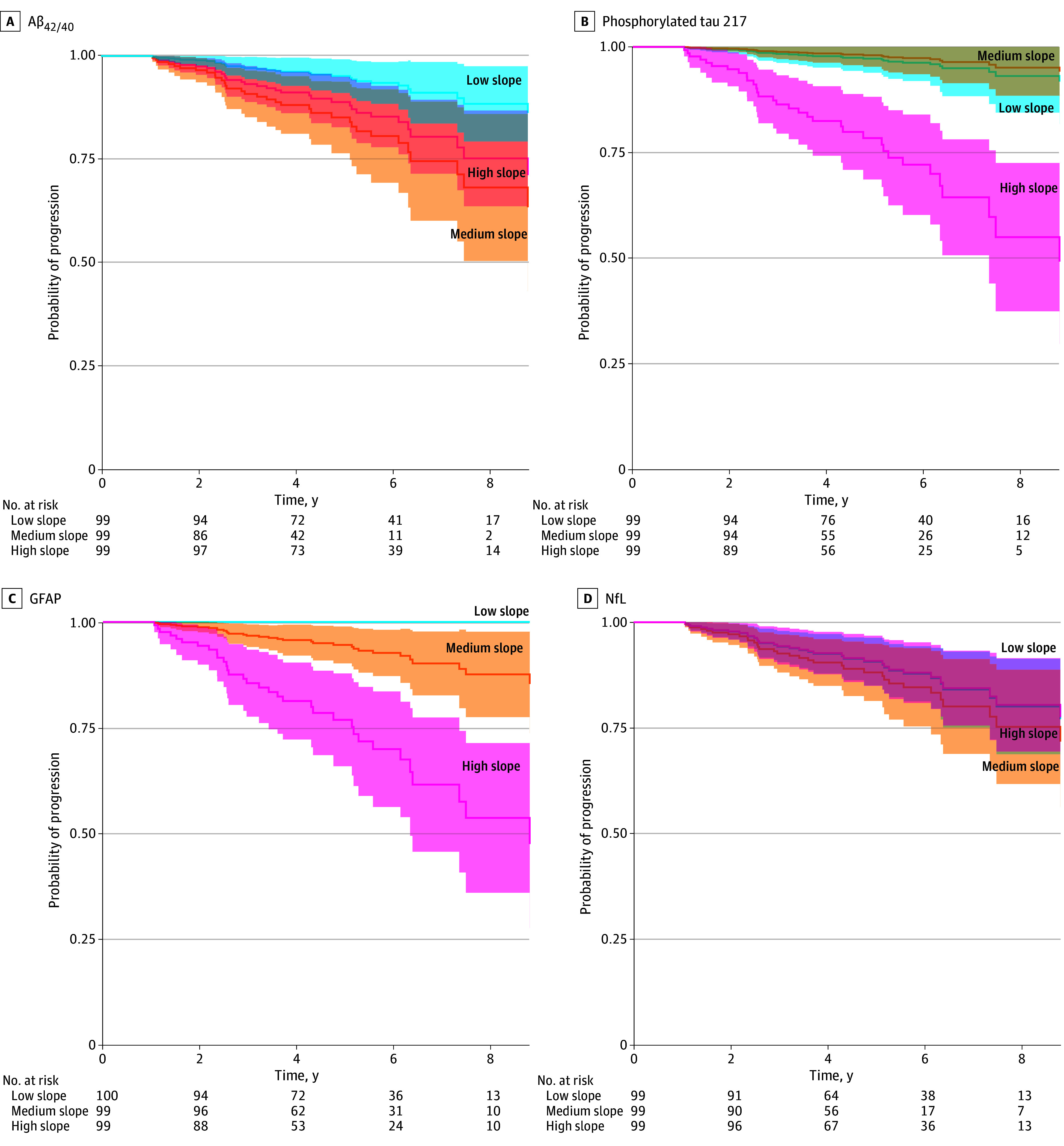
Associations Between Biomarker Slopes and Clinical Progression The figure presents results from Cox proportional hazards regression models showing trajectories of adjusted survival probabilities over time, stratified by tertiles of blood-based biomarker slopes (low, medium, and high). Models were adjusted for age and sex, and survival probabilities reflect the likelihood of progression from subjective cognitive decline to mild cognitive impairment or dementia. Shaded areas indicate 95% CIs. Aβ_42/40_ indicates amyloid-β_42/40_ ratio; GFAP, glial fibrillary acidic protein; NfL, neurofilament light chain.

Lastly, we combined the baseline and slope of all biomarkers and applied elastic net regularization to identify the most prognostic combination of biomarkers. In addition to demographics (age, sex, and baseline MMSE score), the optimal model included baseline Aβ_42/40_ and both baseline and slope values for pTau217 and GFAP, yielding a C index of 0.90 (95% CI, 0.87-0.94). For visualization, participants were stratified into biomarker slope tertiles with the associated risk of progression (Figure 3). Mean associations across all time points in joint models showed that lower Aβ_42/40_ and higher pTau217, GFAP, and NfL were associated with increased risk of progression (eTable 6 in Supplement 1).

### Biomarker Status Change

We dichotomized biomarker values based on each biomarker’s cut-off. Most participants remained stable in their baseline biomarker status, with 102 (34.2%) to 196 (66.0%) remaining stable negative and 52 (17.5%) to 121 (40.7%) remaining stable positive across biomarkers. Among those who were biomarker negative at baseline, 1 in 4 converted to positive during follow-up (Aβ_42/40_: 25 [11.2% of A− individuals], pTau217: n = 50 [24.0%], GFAP: n = 66 [36.7%], and NfL: n = 54 [32.9%]). In contrast, a smaller fraction changed backward (5-18 [1.7%-6.0%] across all biomarkers) or showed intermittent changes (6-23 [2.0%-7.7%]) (eFigure 6 in Supplement 1).

Participants who developed positive pTau217, GFAP, or NfL status showed steeper declines in global cognition (and several other cognitive domains) compared with those who remained biomarker negative (estimate [SD] time × biomarker status β= −0.03 [0.01], *P* = .04 for pTau217; −0.04 [0.01], *P* = .003 for GFAP; and −0.06 [0.02], *P* < .001 for NfL) (eTable 7 and eFigure 7 in Supplement 1), reflecting that these participants did not show cognitive improvement, whereas those who remained biomarker negative did. In contrast, participants who developed positive Aβ_42/40_ did not show steeper cognitive decline. Stable-positive groups for Aβ_42/40_, pTau217, GFAP, and NfL demonstrated steeper cognitive decline in global cognition (and all other cognitive domains) compared with the stable-negative group (estimate [SD] time × biomarker status β = −0.07 ± 0.02, *P* < .001 for Aβ_42/40_; −0.08 [0.02], *P* < .001 for pTau217; −0.08 [0.02], *P* < .001 for GFAP; and −0.08 [0.01], *P* < .001 for NfL).

## Discussion

Our main findings are that longitudinal increases in pTau217, GFAP, and NfL were strongly associated with cognitive decline over time, with pTau217 and GFAP slopes adding prognostic value beyond demographics and baseline biomarker values for progression. In addition, approximately one-fifth of participants converted from biomarker-negative to biomarker-positive status during the 5-year study period. These findings suggest that monitoring these biomarkers can help anticipate future clinical decline, which may be most valuable when early interventions become available.

Among the biomarkers, pTau217 exhibited the most pronounced longitudinal increases in the A+ group, reflecting their association with AD-related pathological changes. In addition, the slope of pTau217 showed the strongest association with cognitive decline over time, consistent with previous literature in cognitively unimpaired individuals without SCD.^14^ Expanding on these findings, our study found that the pTau217 slope provided additional prognostic value for identifying individuals at risk of progression to MCI or dementia. In contrast, Aβ_42/40_ showed differences at baseline between A+ and A− but remained stable over time, reflecting an early plateau in the disease process and limiting its monitoring utility, consistent with prior work.^14^ Moreover, Aβ_42/40_ measured using the Simoa assay may have lower performance compared with other platforms, which could be a potential explanation for the lack of longitudinal findings. Similar to pTau217, GFAP and its changes over time showed strong associations with cognitive decline and clinical progression. NfL also increased in the A+ group, but these changes were less pronounced, and their trajectories did not add prognostic value beyond baseline values for progression. These findings align with the hypothesis that NfL increases modestly in preclinical stages and more steeply as the disease advances. Previous research^14,16,20,21^ has been more inconsistent regarding GFAP and NfL than pTau217. Although several studies observed longitudinal differences between A+ and A- and found associations with cognitive decline,^15,20,21^ others did not.^14,15^ The differences in our findings compared with previous literature may be explained by several factors. First, our sample included individuals with SCD recruited from a memory clinic, a population at higher risk of progression than cognitively unimpaired individuals.^6,8,22^ Second, we used a different assay platform (Simoa) compared with previous studies^14,20^ (eg, Elecsys or in-house immunoassays). Third, we assessed cognitive performance based on a standardized neuropsychological test battery covering 4 domains, allowing for a more comprehensive evaluation. Fourth, our extended follow-up period of up to 15 years (compared with 1-4 years in previous studies^14,20^) enabled the identification of more robust and informative longitudinal biomarker patterns.

One in 5 participants transitioned from pTau217 negative to positive during follow-up. Unlike stable negative participants, who improved in cognition (likely due to learning effects), those transitioning to pTau217 positivity showed no improvements, placing their trajectory between the stable negative and stable positive groups, revealing subtle cognitive decline in line with prior PET-based findings.^23^ Although AD-related pathology is thought to accumulate decades before the onset of dementia, our findings suggest that cognitive changes may begin as early as the transition from an amyloid-negative to amyloid-positive status.

Our study provides novel insights into the longitudinal trajectories of blood-based biomarkers in individuals with SCD. In particular, we highlight that dynamic changes in pTau217, GFAP, and NfL are associated with cognitive decline and clinical progression and that a substantial proportion of individuals with SCD convert to biomarker positivity over time. Individuals with SCD from the memory clinic represent a key population for early detection and intervention because they actively present with cognitive issues while carrying an increased risk of progression compared with cognitively unimpaired individuals. With the advent of disease-modifying treatments, these patients constitute an easily targetable group within SCD trial-ready cohorts and are expected to play an important role in preclinical prevention trials.^24,25^ Our study underscores the potential value of monitoring blood-based biomarker trajectories as a tool for identifying at-risk individuals and detecting clinical changes within these trials. Although current guidelines of the Alzheimer Association Workgroup and International Working Group currently do not recommend biomarker testing in preclinical stages,^8,26^ our findings strengthen the evidence that blood-based biomarkers are reliable indicators of cognitive decline and clinical progression on a group level. Given their non-invasive, cost-effective, and easily repeatable nature, blood-based biomarkers may become reliable enough in the future for individual-level monitoring and prediction in routine clinical care for individuals with SCD, particularly as evidence continues to support the safety of disclosing amyloid status in preclinical stages.^27,28^ However, before such individual-level prediction can be realized, additional longitudinal studies in SCD cohorts are needed to replicate and harmonize findings across research settings.

### Strengths and Limitations

Our study has several key strengths, including a long follow-up period (mean, 4.8 years), the use of multiple blood-based biomarkers, comprehensive cognitive assessment, and a focused examination of individuals with SCD. However, it is important to acknowledge that this study has certain limitations. First, despite the relatively long follow-up, the number of participants progressing to MCI or dementia was modest. This reflects the inherent challenge that, even in extended longitudinal studies, clinical progression in preclinical populations takes substantial time to become observable. Nevertheless, our study provides valuable insight because few other SCD cohorts have been followed up long enough to capture these early clinical transitions. Second, participants were recruited exclusively from a memory clinic, which may introduce selection bias and limit the generalizability of our findings to the broader population. However, this recruitment strategy reflects a clinically relevant population seeking evaluation for cognitive concerns. Third, missing data for several neuropsychological tests posed a challenge, particularly for the Rivermead Behavioral Memory Test, which was introduced after the study commenced. To address this, we applied imputation techniques to estimate missing values, ensuring consistency and enabling the inclusion of this test in the analyses.

## Conclusions

In this prospective cohort study of individuals with SCD, we found that longitudinal measurement of plasma pTau217 and GFAP identified and monitored pathological and clinical progression of AD. These biomarkers show promise for monitoring early disease-related changes before cognitive impairment becomes apparent, and they may prove useful in the future for participant selection and outcome monitoring in preclinical AD trials.

## Notes

Supplement 1. **eAppendix.** Supplementary methods and results **eTable 1.** Baseline levels and longitudinal trajectories of cognition by amyloid status **eTable 2.** Baseline levels and longitudinal trajectories of blood-based biomarkers by amyloid status **eTable 3.** Associations between blood-based biomarkers changes and cognitive performance over time **eTable 4.** Associations between baseline levels of blood-based biomarkers and cognitive performance over time **eTable 5.** Associations between blood-based biomarkers and cognitive domains across all time points **eTable 6.** Associations between blood-based biomarkers and clinical progression across all time points **eTable 7.** Associations between biomarker status change and cognitive trajectories over time **eFigure 1.** Passing-Bablok Regression for Harmonizing Blood-Based Biomarker Values **eFigure 2.** Longitudinal blood-based biomarkers trajectories over time in total group **eFigure 3.** Baseline blood-based biomarkers values stratified by amyloid status **eFigure 4.** Associations between biomarker slopes and cognitive trajectories over time **eFigure 5.** Prognostic performance of blood-based biomarkers for clinical progression **eFigure 6.** Sankey plot of biomarker status change over time per biomarker **eFigure 7.** Associations between biomarker status change and cognitive trajectories over time

Supplement 2.
Data Sharing Statement
