# Supplementary material for: Longitudinal Blood-Based Biomarkers and Clinical Progression in Subjective Cognitive Decline
Source: JAMA Netw Open. 2025 Dec 3;8(12):e2545862. doi: 10.1001/jamanetworkopen.2025.45862 (PMC12676364; doi:10.1001/jamanetworkopen.2025.45862)
Supplement: Supplement 1. — eAppendix. Supplementary methods and results eTable 1. Baseline levels and longitudinal trajectories of cognition by amyloid status eTable 2. Baseline levels and longitudinal trajectories of blood-based biomarkers by amyloid status eTable 3. Associations between blood-based biomarkers changes and cognitive performance over time eTable 4. Associations between baseline levels of blood-based biomarkers and cognitive performance over time eTable 5. Associations between blood-based biomarkers and cognitive domains across all time points eTable 6. Associations between blood-based biomarkers and clinical progression across all time points eTable 7. Associations between biomarker status change and cognitive trajectories over time eFigure 1. Passing-Bablok Regression for Harmonizing Blood-Based Biomarker Values eFigure 2. Longitudinal blood-based biomarkers trajectories over time in total group eFigure 3. Baseline blood-based biomarkers values stratified by amyloid status eFigure 4. Associations between biomarker slopes and cognitive trajectories over time eFigure 5. Prognostic performance of blood-based biomarkers for clinical progression eFigure 6. Sankey plot of biomarker status change over time per biomarker eFigure 7. Associations between biomarker status change and cognitive trajectories over time [file jamanetwopen-e2545862-s001.pdf]

Supplemental Online Content

Trieu C, van Harten AC, van Leeuwenstijn MSA, et al. Longitudinal blood-based biomarkers and clinical progression in subjective cognitive decline. *JAMA Netw Open*. 2025;8(12):e2545862. doi:10.1001/jamanetworkopen.2025.45862

eAppendix. Supplementary methods and results
eTable 1. Baseline levels and longitudinal trajectories of cognition by amyloid status
eTable 2. Baseline levels and longitudinal trajectories of blood-based biomarkers by amyloid status
eTable 3. Associations between blood-based biomarkers changes and cognitive performance over time
eTable 4. Associations between baseline levels of blood-based biomarkers and cognitive performance over time
eTable 5. Associations between blood-based biomarkers and cognitive domains across all time points
eTable 6. Associations between blood-based biomarkers and clinical progression across all time points
eTable 7. Associations between biomarker status change and cognitive trajectories over time
eFigure 1. Passing-Bablok Regression for Harmonizing Blood-Based Biomarker Values
eFigure 2. Longitudinal blood-based biomarkers trajectories over time in total group
eFigure 3. Baseline blood-based biomarkers values stratified by amyloid status
eFigure 4. Associations between biomarker slopes and cognitive trajectories over time
eFigure 5. Prognostic performance of blood-based biomarkers for clinical progression
eFigure 6. Sankey plot of biomarker status change over time per biomarker
eFigure 7. Associations between biomarker status change and cognitive trajectories over time

This supplemental material has been provided by the authors to give readers additional information about their work.

© 2025 Trieu C et al. *JAMA Network Open.*

<u>eAppendix.</u> Supplementary methods and results

Participants

All participants were referred to the memory clinic by their general practitioner, neurologist, or geriatrician for evaluation of cognitive complaints and underwent a standardized baseline workup, including medical history, neurological and physical examination, neuropsychological testing, laboratory assessment, brain MRI, and either CSF biomarker analysis or amyloid PET. Following the standardized work-up, diagnoses were assigned during a multidisciplinary consensus meeting. Participants were labeled with SCD when neuropsychological testing showed normal cognitive functioning and no diagnosis of MCI, dementia, or another neurological or psychiatric disorder known to cause cognitive complaints was present. Participants were enrolled in the Subjective Cognitive Impairment Cohort (SCIENCe) when the main inclusion criteria were met: (1) subjective cognitive complaints in the absence of objective cognitive impairment and (2) age ≥ 45 years.[16] *Exclusion criteria were (1) a diagnosis of MCI or dementia, (2) a current major psychiatric disorder (e.g., major depression, schizophrenia, personality disorder), (3) a neurological disease known to cause cognitive complaints (e.g., Parkinson's disease, epilepsy), (4) HIV infection, (5) abuse of alcohol or other substances, and (6) insufficient proficiency in Dutch.*

Amyloid status

Amyloid PET scans were performed using [^{18}F]florabetapir (n=132), [^{18}F]florabetaben (n=65), [^{18}F]flutemetamol (n=6) or [^{11}C]Pittsburgh compound-B (n=5) tracers on various systems, including Gemini TF PET/CT, Ingenuity TF PET/CT, and Ingenuity PET/MRI (Philips Healtcare, Best, The Netherlands).[7,16] *Amyloid PET scans were visually rated as either "amyloid positive" or "amyloid negative" by trained nuclear medicine physicians. CSF samples were collected by lumbar puncture at the L3/L4, L4/L5 or L5/S1 intervertebral space, using an atraumatic 25-gauge needle. CSF analysis was performed using either Innotest ELISA (Innogenetics-Fujirebio, Ghent, Belgium) or Elecsys*

© 2025 Trieu C et al. *JAMA Network Open.*

electrochemiluminescence immunoassays (Roche Diagnostics GmbH, Mannheim, Germany). Innotest $A\beta_{42}$ *was corrected to account for drift over the years.*[28] *Drift correction was performed by aligning year-specific cutpoints to a uniform reference, resulting in one consistent threshold across all years. Innotest* $A\beta_{42}$ *below 813 pg/ml and Elecsys pTau/Aβ42 ratio above 0.02 were defined as "amyloid positive," while other values were classified as "amyloid negative". When both CSF and amyloid PET were available, the PET result was leading for classification.*

Neuropsychological assessment

For memory, we used the Rey Auditory Verbal Learning Test (RAVLT) - immediate and recall, Visual Association Test (VAT), and Rivermead Behavioral Memory Test (RBMT) - stories and delayed recall.[29-31] *For language, we used the Visual Association Test – naming, semantic fluency, and Boston Naming Test.*[30,32,33] *For attention, we used the Trail-Making Test part A (TMT-A), Stroop task I and II - naming and color naming.*[34,35] *For executive functioning, we used the Trail Making Test part B (TMT-B), Stroop task III – color-word interference, and letter fluency.*[34,35] *Test scores for TMT and Stroop were log transformed, because the data were right-skewed, and subsequently inverted, such that a lower score implies worse performance.*

Blood-based biomarkers

Blood samples were collected by venipuncture in ethylenediaminetetraacetic acid (EDTA) plasma tubes, centrifuged at 1800xg for 10 minutes at room temperature, and subsequently aliquoted into 0.5 mL volumes in polypropylene tubes (Sarsedt, Germany) for storage at -80°C. Samples were thawed at room temperature and centrifuged at 10,000xg for 10 minutes prior to analysis. Analyses of the plasma samples were conducted on the Simoa HD-X analyzer (Quanterix, USA), measuring Aβ1-40, Aβ1-42, GFAP, and NfL with the Simoa™ Neurology 4-plex E Kit (Quanterix) in singlicates and

© 2025 Trieu C et al. *JAMA Network Open.*

pTau217 with the Simoa™ pTau217 kit (Janssen, USA) in duplicates, following the manufacturer's instructions.

© 2025 Trieu C et al. *JAMA Network Open.*

Time-varying LMM

We used linear mixed models with time-varying covariates to assess the association between longitudinal blood-based biomarkers and longitudinal cognition. Each model included terms for the blood-based biomarker and time, with separate models for each biomarker and cognitive domain. The estimates from these time-varying LMMs represent the average association between biomarker levels and cognitive performance across all the time points in the entire study period.

Lower Aβ$_{42/40}$ was associated with worse scores in global cognition (0.135 ± 0.055, $p<0.05$), memory (0.181 ± 0.079, $p<0.05$), and executive functioning (0.163 ± 0.075, $p<0.05$; sTable 2).

Higher pTau217 was associated with worse scores in global cognition (-0.086 ± 0.024, $p<0.01$), memory (-0.128 ± 0.034, $p<0.01$), language (-0.076 ± 0.031, $p<0.05$), and executive functioning (-0.086 ± 0.033, $p<0.05$).

Higher GFAP levels were associated with worse cognition in all cognitive domains, including global cognition (-0.091 ± 0.021, $p<0.01$), memory (-0.105 ± 0.031, $p<0.01$), attention (-0.061 ± 0.027, $p<0.05$), language (-0.083 ± 0.028, $p<0.05$), and executive functioning (-0.097 ± 0.029, $p<0.01$).

Higher NfL were associated with worse global cognition (-0.11 ± 0.028, $p<0.01$), language (-0.15 ± 0.037, $p<0.01$), and executive functioning (-0.102 ± 0.037, $p<0.05$), with additional trends toward worse memory (-0.073 ± 0.04, $p<0.1$) and attention (-0.059 ± 0.035, $p<0.1$).

© 2025 Trieu C et al. *JAMA Network Open.*

Joint models

We used joint models to assess the association between longitudinal blood-based biomarker and the risk of clinical progression to MCI or dementia. Joint modeling was chosen for its specific ability to simultaneously analyze the longitudinal nature of biomarker data and a time-to-event endpoint. The joint models consisted of a longitudinal component to describe the trajectory of the blood-based biomarkers over time, and a survival component to evaluate the time to progression from SCD to MCI/dementia. The longitudinal component was modeled using LMM including terms for time, adjusted for age and sex, with a random intercept and slope for time at the individual level. The survival component was modeled using a Cox proportional hazards model, adjusted for age and sex, where the hazard for progression was estimated using restricted cubic splines. The joint models assessed how blood-based biomarker levels at each time point influenced the hazard of progression, with the mean hazard ratio (HR) reflecting the average effect of the associated risk increase per unit rise in biomarker levels throughout the follow-up period.

Lower $A\beta_{42/40}$ *was associated with an increased risk of MCI/dementia (HR = 0.16, 95% CI: 0.07–0.35, p<0.001). Specifically, a 1 SD lower* $A\beta_{42/40}$ *was associated with an average 6.1-fold increase in the hazard of progression across all time points. Higher pTau217 was associated with an increased risk of progression (HR = 3.64, 95% CI: 2.40–5.82, p<0.001). Higher GFAP was associated with an increased risk of progression to dementia (HR = 1.76, 95% CI: 1.35–2.21, p<0.001). Higher NfL was associated with an increased risk of progression to dementia (HR = 3.01, 95% CI: 1.65–5.48, p<0.001).*

© 2025 Trieu C et al. *JAMA Network Open.*

eTable 1 Baseline levels and longitudinal trajectories of cognition by amyloid status

Cognitive domain	Measure	A-	A+	p-value
Memory	Baseline	0.04 ± 0.04	-0.14 ± 0.10	0.038
	Longitudinal	0.01 ± 0.01	-0.13 ± 0.03	<0.001
Attention	Baseline	0.00 ± 0.05	0.07 ± 0.12	0.520
	Longitudinal	0.02 ± 0.01	-0.04 ± 0.02	0.001
Language	Baseline	-0.02 ± 0.05	0.10 ± 0.10	0.211
	Longitudinal	0.02 ± 0.01	-0.06 ± 0.02	<0.001
Executive Function	Baseline	0.04 ± 0.05	0.02 ± 0.10	0.879
	Longitudinal	0.02 ± 0.01	-0.06 ± 0.02	<0.001
Global Cognition	Baseline	0.01 ± 0.03	0.00 ± 0.08	0.888
	Longitudinal	0.02 ± 0.01	-0.07 ± 0.02	<0.001

The table presents baseline and longitudinal estimates (β ± SE) for each cognitive domain from the linear mixed models. These models were adjusted for age, sex, and education, with values centered around the overall sample mean (age = 61.6 years and sex distribution = 58.4% male, and education level = 5.7 Verhage). For each cognitive domain, values in the A- reflect the intercept for baseline and the annual rate of change (Time estimate) for longitudinal measures. In the A+, baseline values are calculated as the intercept plus the Amyloid_status1 estimate, while longitudinal values represent the annual rate of change as Time plus the interaction term Time * Amyloid_status1. P-values indicate statistical significance between the A+ and A- groups.

© 2025 Trieu C et al. *JAMA Network Open.*

eTable 2 Baseline levels and longitudinal trajectories of blood-based biomarkers by amyloid status

A: Raw values

Biomarker	Measure	A-	A+	p-value
$A\beta_{42/40}$	Baseline	0.064 ± 0.001	0.053 ± 0.002	<0.001
	Longitudinal	0.000 ± 0.000	-0.000 ± 0.000	0.153
pTau217	Baseline	0.04 ± 0.00	0.07 ± 0.00	<0.001
	Longitudinal	0.00 ± 0.00	0.01 ± 0.00	<0.001
GFAP	Baseline	78.17 ± 2.91	109.47 ± 6.50	<0.001
	Longitudinal	4.47 ± 0.38	7.48 ± 0.96	<0.001
NfL	Baseline	13.36 ± 0.54	15.81 ± 1.20	0.023
	Longitudinal	0.57 ± 0.09	1.44 ± 0.23	<0.001

B: Z-scores

Biomarker	Measure	A-	A+	p-value
$A\beta_{42/40}$	Baseline	0.07 ± 0.03	-0.32 ± 0.06	<0.001
	Longitudinal	0.00 ± 0.00	-0.01 ± 0.01	0.154
pTau217 (log)	Baseline	-0.26 ± 0.06	0.85 ± 0.13	<0.001
	Longitudinal	0.06 ± 0.01	0.13 ± 0.02	<0.001
GFAP	Baseline	-0.15 ± 0.06	0.55 ± 0.14	<0.001
	Longitudinal	0.10 ± 0.01	0.17 ± 0.02	<0.001
NfL (log)	Baseline	-0.06 ± 0.05	0.29 ± 0.12	<0.001
	Longitudinal	0.09 ± 0.01	0.13 ± 0.02	0.006

The table presents baseline and longitudinal estimates (β ± SE) for $A\beta_{42/40}$, pTau217, GFAP, and NfL derived from linear mixed-effects models adjusted for age and sex (centered at a mean age of 61.6 years and 58.4 % male). Section A reports raw biomarker values without log transformation or z-scoring, while Section B shows values expressed as z-scores (pTau217 and NfL were log-transformed to accommodate non-normal distributions). For each biomarker, values represent the baseline and annual rate of change (slope) for each group (A–: baseline (intercept) and slope (time estimate); A+: baseline (intercept + Amyloid status estimate) and slope (time estimate + Time*Amyloid status interaction)).

© 2025 Trieu C et al. *JAMA Network Open.*

eTable 3 Associations between blood-based biomarkers changes and cognitive performance over time

Cognitive Domain	$A\beta_{42/40}$ Slope	pTau217 Slope	GFAP Slope	NfL Slope
Global cognition	0.03 ± 0.01*	-0.04 ± 0.01**	-0.02 ± 0.00**	-0.02 ± 0.01*
Memory	0.02 ± 0.02	-0.05 ± 0.01**	-0.03 ± 0.01**	-0.01 ± 0.01
Language	0.04 ± 0.02*	-0.03 ± 0.01*	-0.02 ± 0.00**	-0.03 ± 0.01*
Attention	0.00 ± 0.02	-0.03 ± 0.01**	-0.02 ± 0.00**	-0.02 ± 0.01
Executive function	0.03 ± 0.02.	-0.04 ± 0.01**	-0.02 ± 0.00**	-0.03 ± 0.01*

The table presents the results of linear mixed models analyzing the association between longitudinal changes (slopes) in blood-based biomarkers and cognitive performance over time. The estimates (β ± SE) represent biomarker*time interaction, indicating how biomarker slopes (per 0.05 unit/year increase) are associated with differences in the rate of cognitive change per year. Models were adjusted for baseline levels to isolate the independent effect of the slope over time.

.p < 0.1 (trend)

* p < 0.05

** p < 0.01

eTable 4 Associations between baseline levels of blood-based biomarkers and cognitive performance over time

Cognitive Domain	$A\beta_{42/40}$ Baseline	pTau217 Baseline	GFAP Baseline	NfL Baseline
Global cognition	0.03 ± 0.01**	-0.03 ± 0.00**	-0.03 ± 0.00**	-0.03 ± 0.00**
Memory	0.05 ± 0.02*	-0.03 ± 0.01**	-0.04 ± 0.01**	-0.03 ± 0.01**
Language	0.04 ± 0.02*	-0.03 ± 0.01**	-0.02 ± 0.01*	-0.02 ± 0.01*
Attention	0.02 ± 0.01.	-0.02 ± 0.01*	-0.02 ± 0.01*	-0.03 ± 0.01**
Executive function	0.03 ± 0.01.	-0.02 ± 0.01*	-0.03 ± 0.01**	-0.04 ± 0.01**

The table presents the results of linear mixed models analyzing the association between baseline levels of blood-based biomarkers and cognitive performance over time. The estimates (β ± SE) represent biomarker*time interaction, indicating how baseline biomarker levels (per 1 SD increase) are associated with differences in the rate of cognitive change per year.

.p < 0.1 (trend)

* p < 0.05

** p < 0.01

© 2025 Trieu C et al. *JAMA Network Open.*

eTable 5 Associations between blood-based biomarkers and cognitive domains across all time points

Biomarker	Global cognition	Memory	Attention	Language	Executive function
$A\beta_{42/40}$	0.14 (0.06)*	0.18 (0.08)*	0.07 (0.07)	0.07 (0.07)	0.16 (0.08)*
pTau217 (log)	-0.09 (0.02)**	-0.13 (0.03)**	-0.04 (0.03)	-0.08 (0.03)*	-0.09 (0.03)*
GFAP	-0.09 (0.02)**	-0.1 (0.03)**	-0.06 (0.03)*	-0.08 (0.03)*	-0.1 (0.03)**
NfL (log)	-0.11 (0.03)**	-0.07 (0.04).	-0.06 (0.03).	-0.15 (0.04)**	-0.1 (0.04)*

The table presents the results of linear mixed models examining the relationship between biomarker levels and each cognitive domain. Each biomarker was included as a time-varying covariate, allowing the analysis to assess the average association between biomarker levels and cognitive scores across all time points over the entire study period.

.p < 0.1 (trend)

* p < 0.05

** p < 0.01

© 2025 Trieu C et al. *JAMA Network Open.*

eTable 6 Associations between blood-based biomarkers and clinical progression across all time points

Biomarker	HR (95% CI)
$A\beta_{42/40}$	0.16 (0.07-0.34)**
pTau217	3.62 (2.33-5.52)**
GFAP	1.74 (1.35-2.23)**
NfL	2.89 (1.60-5.01)*

The table presents the results of joint models combining longitudinal biomarker levels and survival data to assess the hazard ratios (HRs) for the risk of progression from subjective cognitive decline (SCD) to mild cognitive impairment (MCI) or dementia. Hazard ratios reflect the increased risk of progression associated with higher biomarker levels measured in standard deviations across all time points.

© 2025 Trieu C et al. *JAMA Network Open.*

eTable 7 Associations between biomarker status change and cognitive trajectories over time

A: $A\beta_{42/40}$

Domain	Measure	Stable-negative	Positive-change	p-value	Stable-positive	p-value
Global cognition	Baseline	0.02 ± 0.04	-0.11 ± 0.09	0.384	0.10 ± 0.07	0.655
	Longitudinal	0.01 ± 0.01	-0.00 ± 0.02	0.460	-0.07 ± 0.02	0.000
Memory	Baseline	0.04 ± 0.04	-0.08 ± 0.15	0.738	0.02 ± 0.09	0.370
	Longitudinal	-0.00 ± 0.01	0.01 ± 0.02	0.574	-0.12 ± 0.04	0.000
Attention	Baseline	0.05 ± 0.06	-0.13 ± 0.13	0.293	0.05 ± 0.10	0.802
	Longitudinal	0.01 ± 0.01	-0.01 ± 0.02	0.329	-0.03 ± 0.02	0.089
Language	Baseline	-0.02 ± 0.05	-0.00 ± 0.12	0.543	0.18 ± 0.07	0.177
	Longitudinal	0.01 ± 0.01	-0.01 ± 0.03	0.274	-0.06 ± 0.04	0.001
Executive functioning	Baseline	0.04 ± 0.05	-0.21 ± 0.16	0.128	0.15 ± 0.09	0.321
	Longitudinal	0.02 ± 0.01	0.01 ± 0.03	0.617	-0.06 ± 0.03	0.001

B: pTau217

Domain	Measure	Stable-negative	Positive-change	p-value	Stable-positive	p-value
Global cognition	Baseline	0.02 ± 0.05	0.03 ± 0.06	0.947	-0.01 ± 0.05	0.730
	Longitudinal	0.03 ± 0.01	0.00 ± 0.01	0.039	-0.07 ± 0.02	0.000
Memory	Baseline	0.07 ± 0.05	-0.01 ± 0.08	0.454	-0.13 ± 0.09	0.030
	Longitudinal	0.01 ± 0.01	-0.01 ± 0.03	0.471	-0.13 ± 0.03	0.000
Attention	Baseline	-0.01 ± 0.07	0.06 ± 0.09	0.580	0.02 ± 0.07	0.663
	Longitudinal	0.03 ± 0.01	-0.00 ± 0.01	0.092	-0.05 ± 0.01	0.000
Language	Baseline	0.01 ± 0.06	0.01 ± 0.06	0.864	0.07 ± 0.06	0.533
	Longitudinal	0.02 ± 0.01	0.01 ± 0.01	0.759	-0.05 ± 0.03	0.001
Executive functioning	Baseline	0.02 ± 0.06	0.05 ± 0.09	0.816	0.03 ± 0.08	0.990
	Longitudinal	0.04 ± 0.01	-0.01 ± 0.02	0.014	-0.06 ± 0.02	0.000

© 2025 Trieu C et al. *JAMA Network Open.*

C: GFAP

Domain	Measure	Stable-negative	Positive-change	p-value	Stable-positive	p-value
Global cognition	Baseline	0.01 ± 0.07	-0.06 ± 0.05	0.244	-0.08 ± 0.06	0.205
	Longitudinal	0.03 ± 0.01	-0.00 ± 0.01	0.003	-0.05 ± 0.02	0.000
Memory	Baseline	0.02 ± 0.07	-0.07 ± 0.07	0.376	-0.04 ± 0.08	0.527
	Longitudinal	0.03 ± 0.01	-0.02 ± 0.02	0.011	-0.10 ± 0.03	0.000
Attention	Baseline	-0.02 ± 0.10	-0.01 ± 0.08	0.882	-0.11 ± 0.10	0.429
	Longitudinal	0.03 ± 0.01	-0.00 ± 0.01	0.023	-0.02 ± 0.01	0.001
Language	Baseline	0.07 ± 0.08	-0.09 ± 0.09	0.105	-0.10 ± 0.08	0.105
	Longitudinal	0.03 ± 0.01	0.01 ± 0.02	0.114	-0.03 ± 0.03	0.026
Executive functioning	Baseline	-0.01 ± 0.09	-0.05 ± 0.07	0.549	-0.04 ± 0.09	0.564
	Longitudinal	0.04 ± 0.01	0.00 ± 0.02	0.021	-0.05 ± 0.02	0.000

D: NfL

Domain	Measure	Stable-negative	Positive-change	p-value	Stable-positive	p-value
Global cognition	Baseline	-0.03 ± 0.07	-0.03 ± 0.07	0.980	-0.02 ± 0.06	0.985
	Longitudinal	0.04 ± 0.01	-0.02 ± 0.02	0.000	-0.04 ± 0.01	0.000
Memory	Baseline	0.03 ± 0.07	-0.08 ± 0.09	0.353	-0.06 ± 0.08	0.338
	Longitudinal	0.03 ± 0.01	-0.03 ± 0.02	0.007	-0.06 ± 0.02	0.000
Attention	Baseline	-0.11 ± 0.10	-0.04 ± 0.10	0.646	0.03 ± 0.08	0.284
	Longitudinal	0.05 ± 0.01	-0.00 ± 0.01	0.008	-0.03 ± 0.01	0.000
Language	Baseline	0.09 ± 0.08	-0.01 ± 0.08	0.800	-0.09 ± 0.08	0.119
	Longitudinal	0.03 ± 0.01	-0.04 ± 0.03	0.014	-0.01 ± 0.01	0.012
Executive functioning	Baseline	-0.11 ± 0.09	-0.01 ± 0.09	0.519	0.07 ± 0.08	0.107
	Longitudinal	0.05 ± 0.01	0.01 ± 0.02	0.011	-0.05 ± 0.01	0.000

The table presents the results of linear mixed models with baseline and longitudinal estimates (β ± SE) for each cognitive domain stratified by biomarker status change, adjusted for age, sex, and education. These models were stratified into three groups: Stable-negative (individuals who stayed below the cut-off value throughout the study period, e.g., $Aβ_{42/40}$ cut-off); Stable-positive (individuals who stayed above the cut-off); and Positive-change (individuals who transitioned from below the cut-off value at baseline to above the cut-off value during the study period). For each cognitive domain, Baseline values reflect the estimate of the intercept from the model, while Longitudinal values reflect the estimate of the Time variable, representing annual rates of change. P-values indicate statistical significance for differences between the Stable-negative group and the respective comparison group (Positive-change or Stable-positive).

© 2025 Trieu C et al. *JAMA Network Open.*

eFigure 1 Passing-Bablok Regression for Harmonizing Blood-Based Biomarker Values

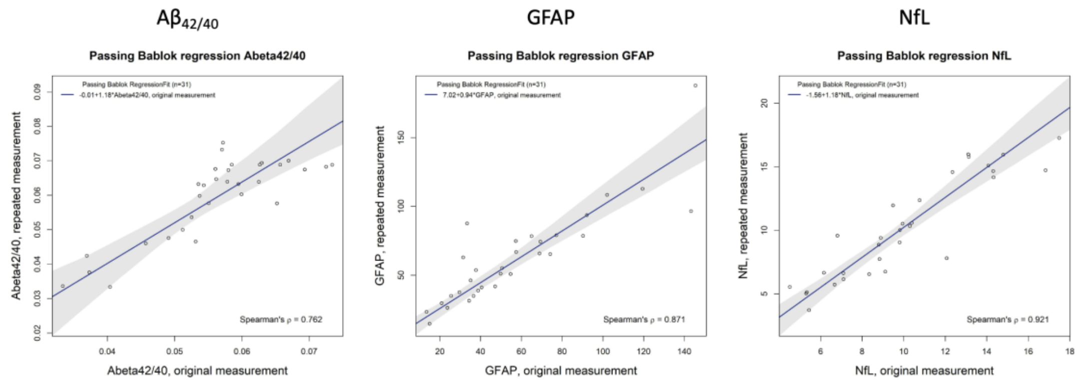

The figure presents the transformation of previously established blood-based biomarker values to the measurement scales used in the current study. Original values were bridged using Passing-Bablok regression to ensure comparability across platforms and assay types. Regression formulas applied for each biomarker are shown in the figure.

eFigure 2 Longitudinal blood-based biomarkers trajectories over time in total group

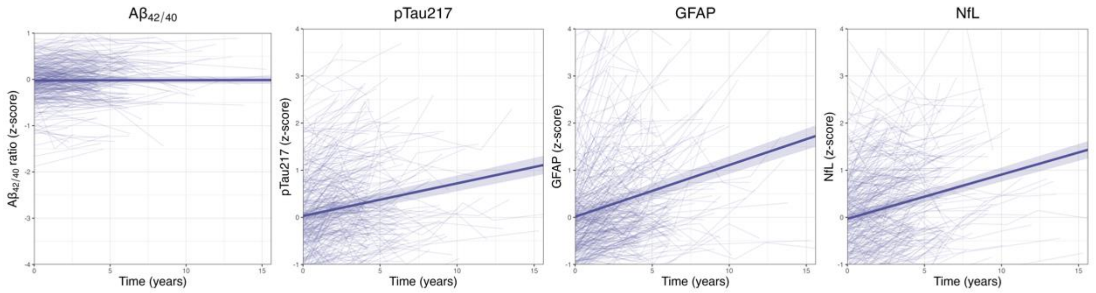

The figure shows the trajectories of blood-based biomarkers over time, presented as z-scores standardized to baseline biomarker values. These figures were adjusted for age and sex, with values centered around the overall sample mean age and sex (i.e., age = 61.6 years, sex distribution = 58.4% male).

eFigure 3 Baseline blood-based biomarkers values stratified by amyloid status

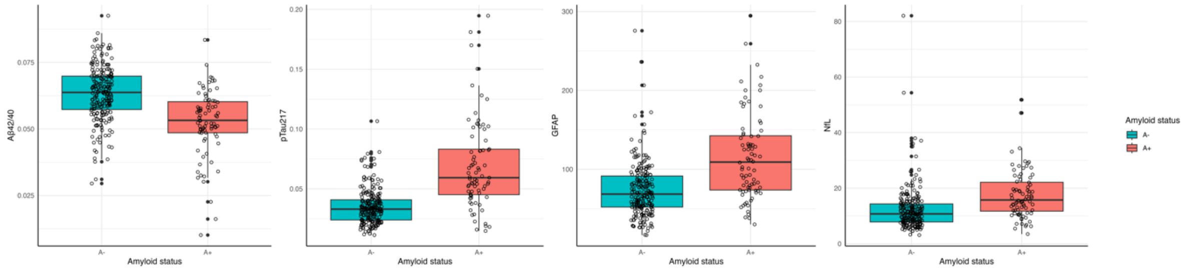

The figure presents the distribution of the blood-based biomarkers at baseline, stratified by amyloid status (A- and A+).

© 2025 Trieu C et al. *JAMA Network Open.*

eFigure 4 Associations between biomarker slopes and cognitive trajectories over time

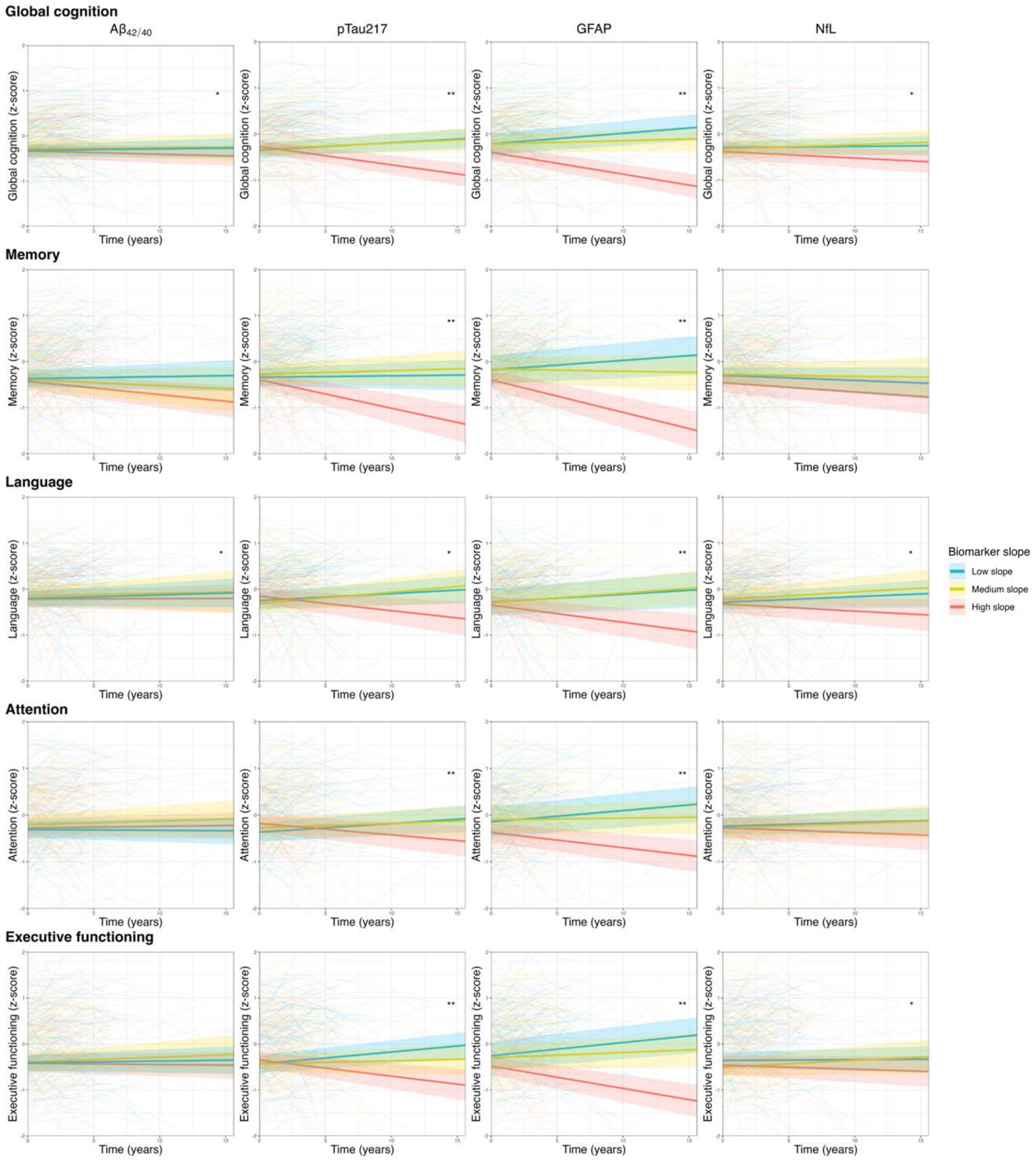

The figure presents results from linear mixed models examining trajectories of cognitive domain scores (Global Cognition, Memory, Language, Attention, and Executive functioning) over time in relation to biomarker slopes. Biomarker slopes were categorized into tertiles (low, medium, high) for visualization. Cognitive scores are expressed as z-scores. P-values shown in the figure represent the biomarker*time interaction, derived from models using continuous biomarker slopes. Models were adjusted for baseline levels (and age, sex and education) to isolate the independent effect of the slope over time.

*p < 0.05, **p < 0.01

© 2025 Trieu C et al. *JAMA Network Open.*

eFigure 5 Prognostic performance of blood-based biomarkers for clinical progression

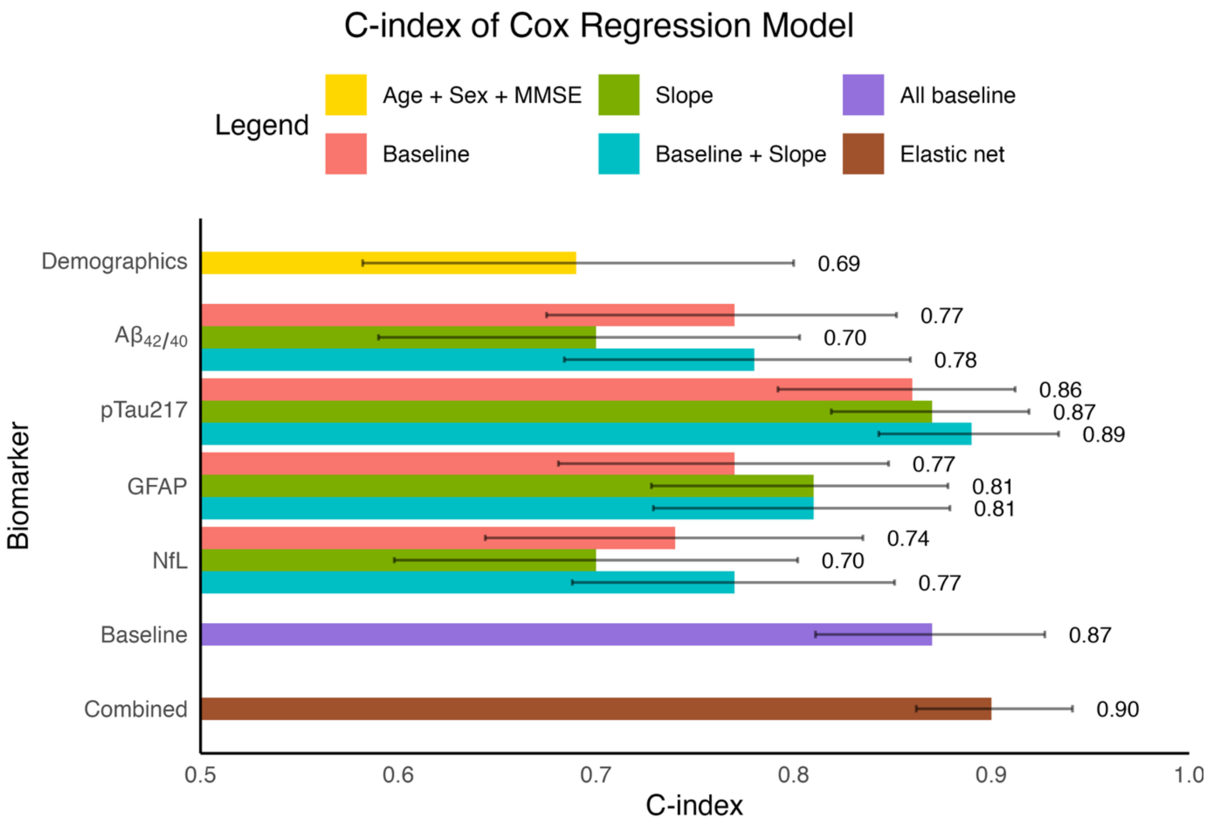

The figure presents the C-index (95 confidence interval) for the blood-based biomarkers from Cox regression models that include (1) demographics (age + sex + baseline MMSE), (2) demographics + baseline biomarker, (3) demographics + slope of biomarker (4) demographics + baseline and slope of biomarker (5) demographics + baseline of all biomarkers*, and (6) demographics + selected biomarkers from elastic net, specifically baseline $A\beta_{42/40}$, baseline + slope pTau217, and baseline + slope GFAP.

eFigure 6 Sankey plot of biomarker status change over time per biomarker

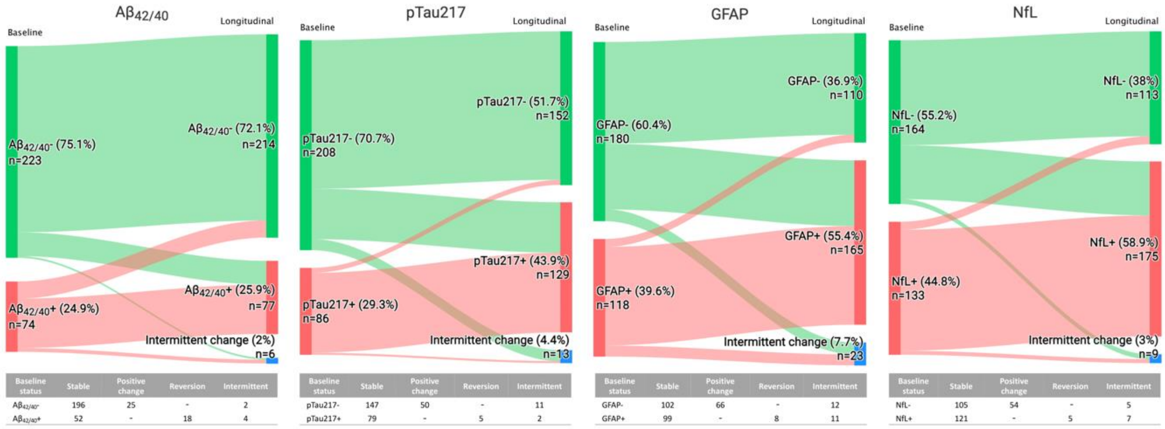

The figure presents the transitions in blood-based biomarker status over time for $A\beta_{42/40}$, pTau217, GFAP, and NfL, visualized using Sankey diagrams. Each Sankey plot represents the proportion of individuals who remain Stable-negative (individuals who remained below the cut-off throughout the study), Stable-positive (individuals who remained above the cut-off), Positive-change (individuals whose biomarker levels transitioned from below to above the cut-off during the study), Negative Change (individuals whose biomarker levels transitioned from above to below the cut-off), and Intermittent Change (individuals whose levels fluctuated above and below the cut-off). The left side of each Sankey plot represents the baseline biomarker status, while the right side represents the biomarker status at the end of the study. The width of each stream corresponds to the number of individuals undergoing a particular transition, illustrating the dynamics of biomarker changes over time.

© 2025 Trieu C et al. *JAMA Network Open.*

eFigure 7 Associations between biomarker status change and cognitive trajectories over time

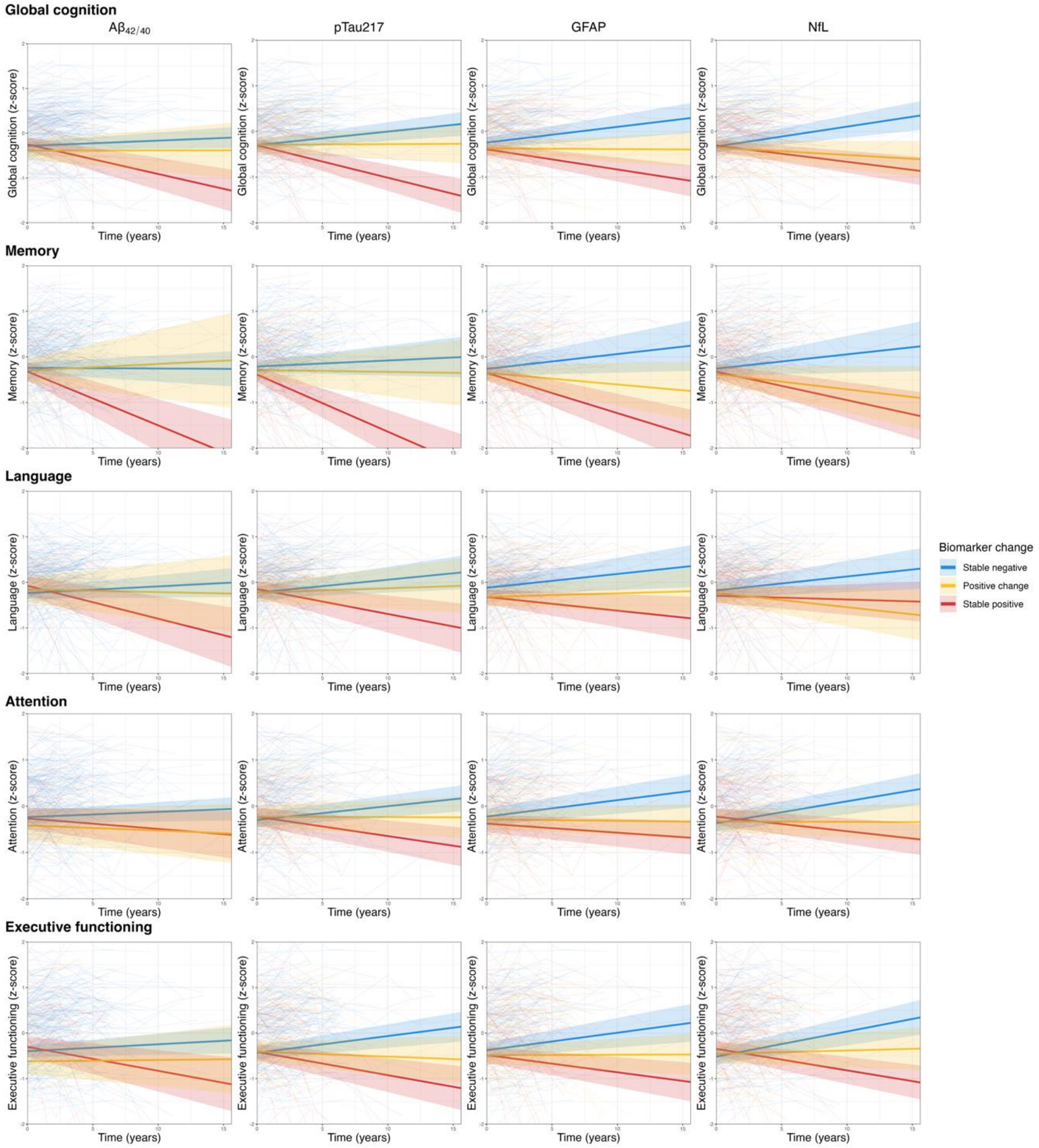

The figure presents results from linear mixed models showing trajectories of cognitive domain scores (Global Cognition, Memory, Language, Attention, and Executive Functioning) over time by biomarker status change. Biomarker status change was categorized into three groups: Stable-negative (blue), which includes individuals who stayed below the cut-off value (e.g., $A\beta_{42/40}$ threshold) throughout the study period; Stable-positive (red), which includes individuals who stayed above the cut-off value throughout the study period; and Positive-change (orange), which includes individuals who transitioned from below the cut-off value at baseline to above the cut-off value during the study period. These models were adjusted for age, sex and education, with values centered around the overall sample mean (age = 61.6 years, sex distribution = 58.4% male, and education level = 5.7 Verhage). Cognitive domain scores are presented as z-scores.

© 2025 Trieu C et al. *JAMA Network Open.*
