# Supplementary material for: Longitudinal Blood-Based Biomarkers and Clinical Progression in Subjective Cognitive Decline
Source: JAMA Netw Open. 2025 Dec 3;8(12):e2545862. doi: 10.1001/jamanetworkopen.2025.45862 (PMC12676364; doi:10.1001/jamanetworkopen.2025.45862)
Supplement: Supplement 2. — Data Sharing Statement [file jamanetwopen-e2545862-s002.pdf]

Data Sharing Statement

Trieu. Longitudinal Blood-Based Biomarkers and Clinical Progression in Subjective Cognitive Decline. *JAMA Netw Open*. Published December 01, 2025.
doi:10.1001/jamanetworkopen.2025.45862

Data
Data available: No

Additional Information
Explanation for why data not available: Data may be shared (anonymized) for purposes of replicating procedures and results within the boundaries imposed by the informed consent and data sharing legislation.
